# Occurrence and Phylogenetic Analysis of Avian Coronaviruses in Domestic Pigeons (*Columba livia domestica*) in Poland between 2016 and 2020

**DOI:** 10.3390/pathogens11060646

**Published:** 2022-06-03

**Authors:** Ewa Łukaszuk, Daria Dziewulska, Tomasz Stenzel

**Affiliations:** Department of Poultry Diseases, Faculty of Veterinary Medicine, University of Warmia and Mazury in Olsztyn, Ul. Oczapowskiego 13, 10-719 Olsztyn, Poland; daria.pestka@uwm.edu.pl (D.D.); tomasz.stenzel@uwm.edu.pl (T.S.)

**Keywords:** coronavirus, pigeons, prevalence, phylogeny, RdRp gene

## Abstract

While disease control in racing pigeons and the potential role of pigeons as vectors transmitting viruses to poultry are of importance, there is still a paucity of data concerning the occurrence of coronaviruses in pigeons. In this study, 215 domestic pigeons were tested for the presence of coronaviral genetic material using the nested PCR method, which revealed 57 positive samples (26.51%). The difference in coronavirus prevalence between young and adult pigeons (34.34% and 19.83%, respectively) has been found statistically significant. In contrast, no statistically significant difference has been demonstrated between the prevalence in symptomatic and asymptomatic birds, leaving the influence of coronavirus presence on pigeon health uncertain. Phylogenetic analysis of the RdRp gene fragment allowed us to assign all the obtained strains to the *Gammacoronavirus* genus and *Igacovirus* subgenus. The phylogenetic tree plotted using the ML method revealed that those sequences formed a group most similar to pigeon coronavirus strains from China, Finland, and Poland, and to a single strain from a common starling from Poland, which suggests wide geographical distribution of the virus and its possible transmission between various species.

## 1. Introduction

Coronaviruses from the *Orthocoronavirinae* subfamily, i.e., those representing the *Deltacoronavirus* and *Gammacoronavirus* genera, are relatively widespread across bird species [1]. Viruses from the *Gammacoronavirus* genus are responsible for infections of poultry and wild birds, although the latter are known to also be infected by viruses from the *Deltacoronavirus* genus [2,3]. Viruses from both these genera have been found in pigeons belonging to the *Columba livia* species [4,5]. Coronaviruses are considered prevalent disease factors in birds. Their genetic material, detected in the gastrointestinal tract, is considered a putative cause of health problems affecting this system, collectively referred to as enteropathies [6,7]. Racing pigeons are also afflicted by these diseases, which can severely impair their racing performance. The growing popularity of pigeon racing puts a demand on veterinary medicine specialists to extend their knowledge to also include an understanding of the control of viral infections, which are suspected of causing enteropathies. From the practical point of view, racing pigeon breeding is a combination of mass breeding (because birds are kept in flocks) with the breeding of companion animals, hence, the market value of an individual bird can be very high, depending on its pedigree, phenotype, and racing results [8]. Keeping birds in flocks contributes to the incidence of infectious diseases being the main health problem in this population. A potential high market value of an individual racing pigeon makes controlling infectious diseases with methods known in mass breeding impossible to apply—eradication of whole lines or flocks is not routinely performed. However, according to some legal regulations, i.e., The Act of 11 March 2004 on the Protection of Animal Health and Combating Infectious Animal Diseases, issued by the Sejm of the Republic of Poland, domestic pigeons are considered poultry [9]. As stated in the OIE Terrestrial Animal Health Code, “poultry” means “all birds reared or kept in captivity for the production of any commercial animal products or for breeding for this purpose” [10]. Pigeons of different utilities are kept worldwide, being reared for meat production in many countries, although the main part of the population consists of either racing or ornamental birds [8,11,12,13,14,15]. Two types of domestic pigeons are kept in Poland: racing carrier pigeons (the population is about 5 million breeding and racing birds altogether, and about 5 million young pigeons are reared each year) and exhibition ornamental pigeons (the population is about 300,000 breeding birds, and about 280,000 young pigeons are reared each year). There are also amateur breeders who are not affiliated with any organization, so the number of birds in these breeding facilities is difficult to estimate. During races, racing pigeons travel distances as long as thousands of kilometers, which makes them potential vectors of diseases, especially when a carrier of a poultry pathogen lands to rest in the vicinity of a poultry farm.

To date, coronaviruses genetically similar to infectious bronchitis virus (IBV) have been confirmed to occur in pigeons. Detection of viral strains with a nucleotide sequence of the S1 fragment nearly identical to that of Massachusetts and Connecticut vaccine serotypes in asymptomatic feral pigeons suggests the possible transfer of IBV between pigeons and chickens [16]. While IBV and related avian coronaviruses are most often associated with respiratory tract infections, several cases of the formation of different variants changing the tropism to the tissues have been reported. Replication at non-respiratory epithelial surfaces is considered a cause of the pathology of kidneys, oviduct, testes, and alimentary tract of chickens [17,18]. Coronaviruses responsible strictly for inducing enteropathies have also been detected, e.g., the turkey coronavirus (TCoV), causing an enteric disease manifested as a lack of appetite, weight loss, and excretion of watery droppings in turkey flocks [19]. To date, there are no records of TCoV occurrence in pigeons, although infections of a similar clinical picture and unknown etiology have been observed in their population. In addition, to date there is no research describing coronavirus isolation in pigeons affected by the gastrointestinal disease.

Avian coronaviral infections have been a focus of interest of researchers for the past few years; however, most studies addressing this issue concern poultry kept in the intensive production system [20]. Most recently, a few studies have been undertaken to determine the prevalence of coronaviruses in wild birds [2,3,21,22,23,24,25,26,27]. However, little is known about the prevalence of viruses in domestic pigeons, which could represent a link between wild birds and poultry, allowing the viruses to spread. Therefore, we conducted the research to answer questions about whether avian coronaviruses occur in the population of domestic pigeons in Poland, what their prevalence is, and what their genetic relationship with other known avian coronaviruses may be. Our study also aimed to determine whether there is a link between the occurrence of coronaviruses in pigeons and enteropathies.

## 2. Results

### 2.1. Prevalence of Coronaviral Genetic Material in Investigated Pigeons

Fifty-seven out of the two hundred fifteen studied samples (26.51%) tested positive for coronaviral genetic material. Twenty-three out of one hundred sixteen samples (19.83%) and thirty-four out of ninety-nine samples (34.34%) derived respectively from adult and young birds were found to be positive. Seventeen out of eighty-seven samples (19.54%) acquired from asymptomatic pigeons and forty out of one hundred twenty-eight samples (31.25%) acquired from symptomatic pigeons turned out to be positive (Table 1). Additionally, out of 38 tissue samples derived from dead birds, and out of 177 cloacal swab samples collected from live birds, 5 (13.16%) and 52 (29.38%) have been found positive, respectively.

Despite the very distinct difference in the prevalence of coronaviral genetic material between symptomatic and asymptomatic pigeons, the only statistically significant difference has been found in coronavirus prevalence between adult and young birds (χ^2^ = 5.78, *p* = 0.0162). Differences between asymptomatic birds and symptomatic birds, asymptomatic adult birds and symptomatic adult birds, asymptomatic young birds and symptomatic young birds, asymptomatic adult birds and asymptomatic young birds, and between symptomatic adult birds and symptomatic young birds have proven to be statistically insignificant (χ^2^ = 3.65, *p* = 0.0562; V^2^ = 0.05, *p* = 0.8199; χ^2^ with Yates correction = 0.63, *p* = 0.4269; χ^2^ with Yates correction = 0.03, *p* = 0.8623; and χ^2^ = 3.21, *p* = 0.0732; respectively). There was also a significant difference (χ2 = 4.22, *p* = 0.0398) in coronavirus prevalence between live and dead birds.

### 2.2. Phylogenetic Analysis

**Figure 1:**
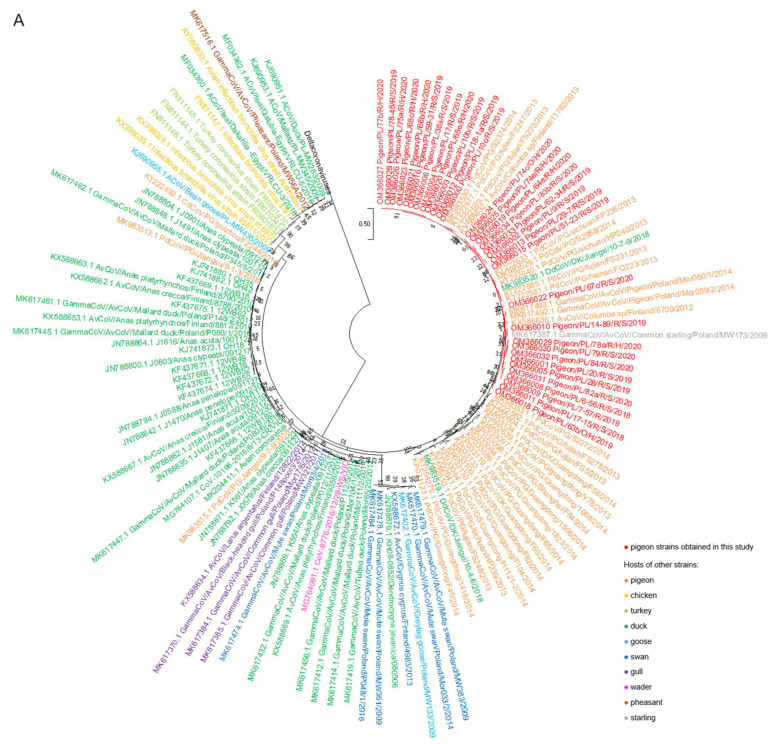

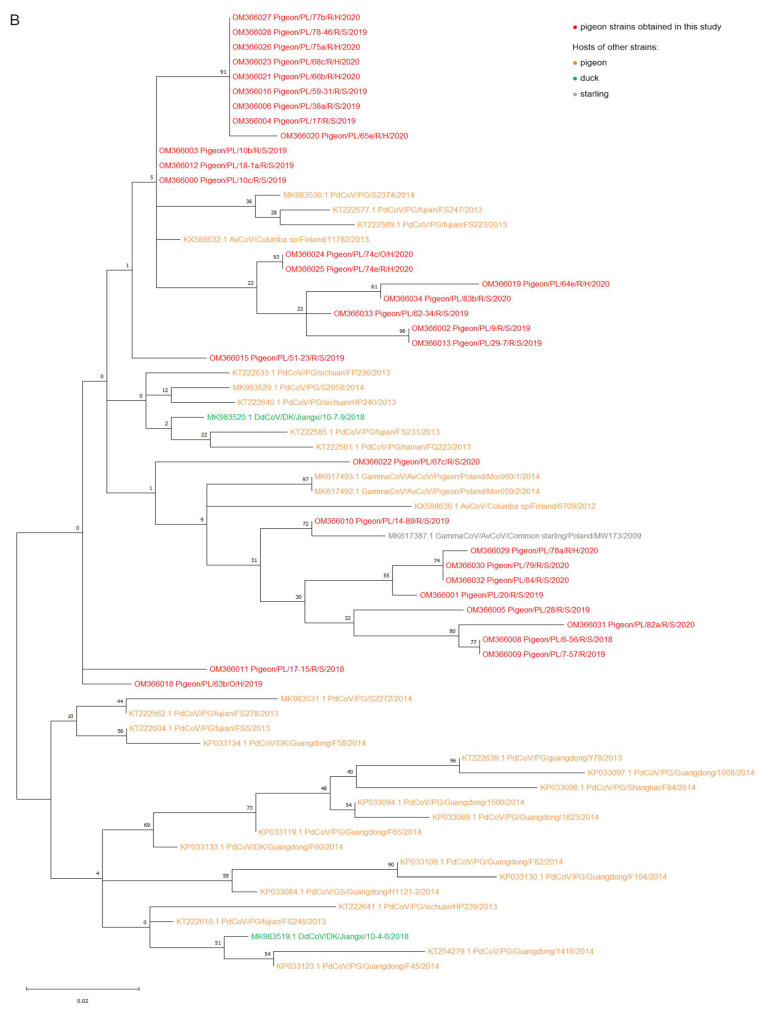
(**A**) The phylogenetic tree of RdRp gene nucleotide sequences (440 bp) of 101 various avian coronavirus strains available in the GenBank database and 32 pigeon coronavirus strains obtained in this study. (**B**) The phylogenetic subtree consisting of the “pigeon-dominant” strains. The trees were inferred in Molecular Evolutionary Genetics Analysis software, version 11 (MEGA 11; https://megasoftware.net/, accessed on 17 December 2021), by using the maximum likelihood method and Tamura 3-parameter model with the highest log likelihood (−4391.32) [28,29]. The initial tree for the heuristic search was obtained automatically by applying the neighbor-joining algorithm to a matrix of pairwise distances estimated using the Tamura 3 parameter model. A discrete gamma distribution was used to model evolutionary rate differences among sites (five categories (+*G*, parameter = 0.5384)). The bootstrap method was used in a number of 1000 repetitions. The trees are drawn to scale, with branch lengths measured in the number of substitutions per site. All positions containing gaps and missing data were eliminated. The coronavirus strains obtained in this study and from the NCBI database are marked with different colors.

The results of a phylogenetic analysis of a 440 bp RdRp gene fragment revealed that all tested strains belonged to the *Gammacoronavirus* genus and *Igacovirus* subgenus. According to the established tree, Polish sequences of pigeon coronaviruses formed one separate group most closely related to the coronavirus strains obtained from domestic pigeons from China (GenBank accession numbers KP033084.1, KP033089.1, KP033094.1, KP033097.1, KP033098.1, KP033109.1, KP033119.1, KP033123.1, KP033130.1, KP033133.1, KP033134.1, KT222561.1, KT222562.1, KT222577.1, KT222585.1, KT222589.1, KT222604.1, KT222610.1, KT222633.1, KT222639.1-KT222641.1, KT254279.1 and MK983529.1-MK983531.1) and feral pigeons from Finland and Poland (GenBank accession numbers KX588632.1 and KX588636.1; and MK617492.1-MK617493.1, respectively) (Figure 1A,B). Two strains acquired from ducks from China (GenBank accession numbers MK983519.1 and MK983520.1) also were positioned closely to Polish pigeon strains on the tree. Another strain that is very closely related to Polish strains according to the phylogenetic tree was the coronavirus obtained from common starling from Poland (GenBank accession number MK617387.1).

## 3. Discussion

Our study revealed that over a quarter of the samples tested contained coronaviral RNA. However, quite high coronavirus prevalence values have also been found in other avian species to date (i.e., 26.7% and 20.5% prevalence in wild ducks in Australia and Sweden, respectively, 13.0% in herons in Cambodia, and 9.7% in domestic turkeys in Poland) [7,21,24,25]. Therefore, one may deduce that coronaviruses are generally prevalent in various species of birds, and such high prevalence in pigeons may result from the relatively high incidence of subclinical infections occurring in domestic pigeons. We do not include the prevalence of coronaviruses in chickens in our comparison, because commercial chickens are routinely vaccinated with IBV vaccines, which results in exceptionally high prevalence in coronavirus surveillances (even as high as 94.4%) [30].

The majority of positive samples were acquired from young pigeons, which has been proved to be statistically significant. The higher prevalence of coronaviruses in the group of young pigeons might be caused by their not yet fully developed immune system. It could also be due to the stress related to leaving the parental nest and, in the case of racing pigeons, to racing training and transport. This phenomenon has been confirmed for pigeon circovirus, pigeon herpesvirus, and pigeon rotavirus, and could be likely also in the present study [31,32].

Despite the lack of statistical differences between the prevalence of coronaviruses in asymptomatic pigeons and pigeons with selected clinical symptoms, a certain trend was noticeable also in young birds, because the prevalence of the genetic material of coronaviruses was about 70% higher in young pigeons showing selected disease symptoms than in asymptomatic young pigeons. Moreover, higher prevalence was determined in the samples collected from live than dead birds, and this finding has been proven statistically significant. Therefore, one may assume that the coronavirus infection either may not be related to the birds’ death, might have overlapped with another health problem, or was a part of a complex infection with other pathogens. However, this finding might not be entirely reliable due to the small number of samples collected from dead birds. We have not investigated the samples for the presence of genetic material of other known viruses pathogenic to pigeons. Due to some contradictions in the results obtained, it cannot be clearly stated whether pigeon coronaviruses are pathogenic to this bird species and whether they can induce enteric problems. The above analysis is also hindered by the very common asymptomatic infections in pigeons, which was also confirmed in our current research. The ideal solution to confirm the potential role of pigeon coronaviruses as triggers of enteropathies would be an experimental infection with laboratory-propagated isolates of these viruses, which would allow for meeting Koch’s postulates. Unfortunately, these assumptions cannot be addressed because other research indicates great difficulties with the cultivation of these viruses in laboratory conditions, which makes it impossible to obtain a pure isolate for experimental inoculation [33,34].

Analysis of the RdRp gene fragment, which is a conserved region of a coronaviral genome used for classification, allowed us to assign all strains obtained in the current research to the *Gammacoronavirus* genus. Only one case of discovery of a virus from the *Deltacoronavirus* genus in pigeon was documented among all surveillances to date [5]. Further research would be useful to confirm or exclude the presence of deltacoronaviruses in the population of Polish pigeons.

Analysis of the phylogenetic tree revealed that pigeons were hosts of the coronavirus typical of this bird species, which is confirmed by the close position of the Polish strains and those originating from pigeons from other European countries and China on the phylogenetic tree. The above finding was consistent with the results reported by Zhuang et al., who investigated 170 pigeon coronavirus sequences obtained in China in the years 2013, 2014, and 2018, constituting one large group of strains, acquired mainly from pigeons, called pigeon-dominant coronavirus [33]. The similarity of the coronaviruses detected in our research to those obtained in China may be due to the fact that Chinese researchers studied coronavirus strains acquired from pigeons originating from different breeding facilities. It should be mentioned that Asian countries are currently among the most engaged in pigeon racing, and that breeders from China import carrier pigeons from all over Europe [35]. This allows various viruses to spread to other bird populations, and is a potential explanation of findings from our study. However, explicit confirmation of this theory is difficult due to the small number of pigeon coronavirus sequences available in the GenBank database.

Zhuang et al. also recognized several smaller groups, associated with sequences derived mainly from wild birds and domestic waterfowl, closely related to pigeon strains. According to our phylogenetic tree, two strains acquired from ducks from China were closely related to Polish pigeon strains. These two strains were identified by the researchers as pigeon-dominant, suggesting that such strains are most commonly isolated from pigeons and have a very high resemblance to pigeon strains [26,33].

Interestingly, the pigeon coronavirus strains from Poland are positioned closely to the coronavirus obtained from the common starling on the phylogenetic tree. Although we are unable to provide a clear explanation for their close relationship, the possible contact of racing pigeons with wild birds during free flights in the areas surrounding lofts might be a trigger of cross-infection. Moreover, the very low genetic identity of the strains studied with IBV and turkey coronavirus sequences indicates that those viruses definitely belong to different species.

Recently, coronaviral infections have been receiving a significant amount of attention from both the public and researchers. Despite the very large genome, viruses belonging to the *Coronaviridae* family generally have very low mutation rates compared to other RNA viruses because of the presence of exonuclease in nsp14 protein, which remarkably increases replication fidelity by the removal of misincorporated nucleotides [36,37,38]. However, the gene coding the spike protein is considered a very variable region of the coronaviral genome due to frequent point mutations and recombination events within it, which is exceptionally significant considering the spike protein promotes binding to host receptors. In this way, through alterations in spike gene, coronaviruses may change cell and tissue tropism, increase virulence, or adapt to new hosts. Moreover, nonhomologous recombination is possible with different coronavirus genera, other viruses, and even hosts, which further increases the potential for new strains to emerge [39,40]. While gammacoronaviruses and deltacoronaviruses are usually associated with infections of avian species, there is also evidence of their occurrence in mammals [41,42]. Considering all of the above, concerns about the possibility of the appearance of highly pathogenic variants threatening poultry, which could cause large losses especially in the intensive production systems, are entirely reasonable. Domestic pigeons, as animals that are free-flying and habituated to humans, have the potential for both being viral reservoirs and for transmitting various pathogens between poultry facilities and wild birds. For example, cases of detection of viruses pathogenic to poultry in pigeons have been documented for CIAV, PPMV-1 and HPAIV subtype H5N1, among others [43,44,45,46]. Due to the paucity of data about the genetic diversity and distribution of coronaviruses, monitoring pigeons for potentially pathogenic factors is of undeniable importance. Further research seems advisable to better assess large-scale prevalence, variability, and the role of coronaviruses in pigeons pathology.

## 4. Materials and Methods

### 4.1. Ethical Statement

No ethical approval was required for this study, as the samples consisted of cloacal swabs collected from birds being patients of the Department of Poultry Diseases, Faculty of Veterinary Medicine, University of Warmia and Mazury in Olsztyn, Poland, during standard diagnostic procedures, for which the consent of their owners was obtained. No other manipulations were performed on live animals. Tissue samples were collected during necropsy of dead birds delivered to the Department of Poultry Diseases.

### 4.2. Sample Collection

**Figure 2:**
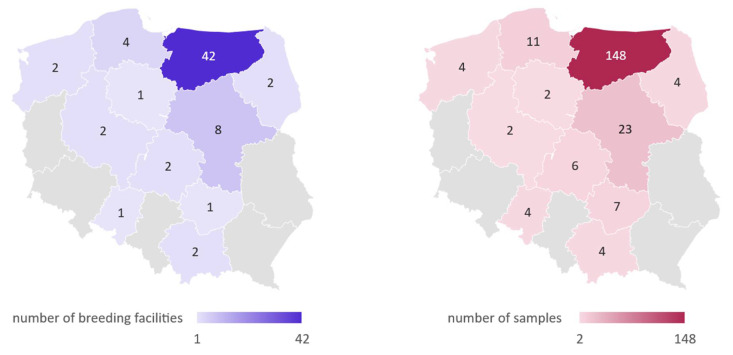
The geographical distribution of pigeon samples collected in this study.

Experimental material was collected from 215 domestic pigeons originating from 67 breeding facilities located in multiple regions of Poland between January 2016 and August 2020 (Figure 2). The pigeons were provided by pigeon fanciers collaborating with the Department of Poultry Diseases, Faculty of Veterinary Medicine, University of Warmia and Mazury in Olsztyn, Poland. Out of all birds, 116 were over 4 months of age and have been classified as adult, while 99 were under 4 months of age and classified as young. Pigeons not showing clinical signs of any disease (n = 87) were classified as asymptomatic, and birds classified as symptomatic exhibited at least one of the following clinical signs: polyuria, greenish diarrhea, regurgitation, water in the crop, lack of appetite, apathy, poor growth, or cachexia (n = 128). In the case of deaths noticed in pigeon flocks, birds with the following post-mortem lesions were included in the study: liquid content in the bowels, hyperemia of kidneys, or kidney swelling. Cloacal swab samples (n = 177) were collected from live birds, and kidney or intestine sections (n = 38) were collected from dead individuals. Samples were suspended in 400 µL of a Fenozol solution (A&A Biotechnology, Gdańsk, Poland) and stored in −20 °C until analyzed.

### 4.3. Molecular Detection of Coronaviruses

The total RNA was extracted from the samples suspended in Fenozol solution using a Total RNA Mini Plus kit (A&A Biotechnology, Gdańsk, Poland) in accordance with the manufacturers’ protocol, and eluted in 50 μL of RNase-free water. Purity and concentration of the obtained RNA were measured with a NanoDrop 2000 Microvolume Spectrophotometer. cDNA was generated through reverse-transcribing 2 μL of eluted RNA using a High-Capacity cDNA Reverse Transcription Kit (Applied Biosystems, Waltham, MA, USA). 2 µL of the acquired cDNA were then used for nested PCR for the RNA-dependent RNA polymerase—gene (RdRp gene) fragment in the final volume of 20 µL. A HotStarTaq Plus Master Mix Kit (Qiagen, Hilden, Germany) was used to prepare the reaction mixture. Two rounds of PCR were performed according to the method used by Chu et al., in a MasterCycler Pro thermocycler (Eppendorf, Hamburg, Germany) [21]. The second primer set used in this method amplifies a 440 bp fragment of the RdRp gene, corresponding to position 14193–14633 nucleotides in the avian infectious bronchitis virus genome (GenBank accession number NC_001451.1). This method of analysis of samples for the presence of coronaviral genetic material, introduced in 2011, has been used in numerous studies regarding coronavirus occurrence and their genetic relationship in wild birds [22,23,25,26]. After carrying out electrophoresis in 2% agarose gel and reading the results with a GelDoc gel imaging system (Bio-Rad, Hercules, CA, USA), the positive samples were excised from the gel and purified using a Clean-Up kit (A&A Biotechnology, Gdańsk, Poland). Sanger dideoxy sequencing of the selected samples was then carried out in Genomed S.A. laboratory (Warsaw, Poland). Sequencing was performed in two directions with the primers from second step of the nested PCR as sequence primers.

### 4.4. Sequence and Phylogenetic Analysis

The sequences obtained were assembled into the contigs in the SeqMan Pro software version 14.1 (DNASTAR, Madison, WI, USA) and manually checked for sequencing errors using the same software. These sequences, compiled with RdRp gene sequences of other avian coronaviruses retrieved from the GenBank database (NCBI) using the BLAST tool, were aligned with the MUSCLE method using Molecular Evolutionary Genetics Analysis software, version 11 (MEGA 11) [29]. All aligned sequences were trimmed at the same 5′ and 3′ ends, resulting in fragments of around 440 bp in length, and used for further analyses. Then the phylogenetic analysis was performed with MEGA 11, using the statistical method indicated by the Find Best DNA/protein models function, which was maximum likelihood, with the Tamura 3-parameter substitution model, gamma distributed pattern with 5 discrete gamma categories, and complete deletion as the method for gaps or missing data treatment [28]. To estimate the credibility of the obtained topologies on the phylogenetic tree, the bootstrap method was used in a number of 1000 repetitions. GenBank sequences of the identical strains have been removed from the tree to provide readability.

### 4.5. Nucleotide Sequence Accession Numbers

Viral sequences obtained in this study and used in this paper have been deposited in GenBank under accession numbers OM366000 to OM366006, OM366008 to OM366013, OM366015, OM366016, and OM366018 to OM366034.

### 4.6. Statistical Analysis

The data obtained has been subjected to a statistical analysis using Statistica 13.3 software (Statsoft, Cracow, Poland). The chi-square test (χ^2^), chi-square test with Yates correction, and V-square test (V^2^) have been used to determine the significance of differences in selected parameters, depending on the expected values in the compared groups. Differences were considered significant with *p* < 0.05.

## Figures and Tables

**Table 1:** The prevalence of coronavirus genetic material in the investigated pigeons. n represents the total number of samples, n+ represents the samples positive for coronaviral genetic material. The values in the same column with different superscripts (a, b) differ significantly with *p* < 0.05 in χ^2^ test.

	CoV		
Group of Pigeons	n	n+	%
Adult	116	23	19.83 ^a^
Young	99	34	34.34 ^b^
Asymptomatic	87	17	19.54
Symptomatic	128	40	31.25
Adult asymptomatic	73	14	19.18
Adult symptomatic	43	9	20.93
Young asymptomatic	14	3	21.43
Young symptomatic	85	31	36.47

## Data Availability

The genome sequences generated in this study were submitted to the GenBank database (https://www.ncbi.nlm.nih.gov/genbank/) (accessed on 26 January 2022) under accession numbers OM366000 to OM366006, OM366008 to OM366013, OM366015, OM366016, and OM366018 to OM366034.
